# In Vivo Chemical Screening in Zebrafish Embryos Identified FDA-Approved Drugs That Induce Differentiation of Acute Myeloid Leukemia Cells

**DOI:** 10.3390/ijms25147798

**Published:** 2024-07-16

**Authors:** Xiaona Wei, Wei Wang, Qianlan Yin, Hongji Li, Abrar Ahmed, Rahat Ullah, Wei Li, Lili Jing

**Affiliations:** 1 Engineering Research Center of Cell & Therapeutic Antibody, Ministry of Education, School of Pharmacy, Shanghai Jiao Tong University, Shanghai 200240, China; weixiaona@sjtu.edu.cn (X.W.); yql0218@sjtu.edu.cn (Q.Y.); toyli2233@sjtu.edu.cn (H.L.); abrar13@bu.edu (A.A.); rahat_2020@sjtu.edu.cn (R.U.); 2 Nanozyme Medical Center, School of Basic Medical Sciences, Zhengzhou University, Zhengzhou 450001, China; wangweivivi@zzu.edu.cn; 3 Core Facility and Technical Service Center, School of Pharmacy, Shanghai Jiao Tong University, Shanghai 200240, China

**Keywords:** acute myeloid leukemia, ethacrynic acid, IL-17/MAPK, myeloid differentiation, all-trans retinoic acid, zebrafish

## Abstract

Acute myeloid leukemia (AML) is characterized by the abnormal proliferation and differentiation arrest of myeloid progenitor cells. The clinical treatment of AML remains challenging. Promoting AML cell differentiation is a valid strategy, but effective differentiation drugs are lacking for most types of AML. In this study, we generated Tg(*drl:hoxa9*) zebrafish, in which *hoxa9* overexpression was driven in hematopoietic cells and myeloid differentiation arrest was exhibited. Using Tg(*drl:hoxa9*) embryos, we performed chemical screening and identified four FDA-approved drugs, ethacrynic acid, khellin, oxcarbazepine, and alendronate, that efficiently restored myeloid differentiation. The four drugs also induced AML cell differentiation, with ethacrynic acid being the most effective. By an RNA-seq analysis, we found that during differentiation, ethacrynic acid activated the IL-17 and MAPK signaling pathways, which are known to promote granulopoiesis. Furthermore, we found that ethacrynic acid enhanced all-trans retinoic acid (ATRA)-induced differentiation, and both types of signaling converged on the IL-17/MAPK pathways. Inhibiting the IL-17/MAPK pathways impaired ethacrynic acid and ATRA-induced differentiation. In addition, we showed that ethacrynic acid is less toxic to embryogenesis and less disruptive to normal hematopoiesis than ATRA. Thus, the combination of ethacrynic acid and ATRA may have broader clinical applications. In conclusion, through zebrafish-aided screening, our study identified four drugs that can be repurposed to induce AML differentiation, thus providing new agents for AML therapy.

## 1. Introduction

Acute myeloid leukemia (AML) is a diverse form of primary hematopoietic neoplasm originating largely from cells committed to the myeloid lineage of cellular development [1]. It commonly arises as a de novo malignancy, leading to the severe impairment of the hematopoietic system [2,3]. On average, 10–40% of patients with newly diagnosed AML will fail to attain complete remission (CR) with intensive chemotherapy. Additionally, up to 50% of the patients who initially achieve CR will subsequently develop relapsed AML [4]. Allogeneic hematopoietic stem cell transplantation (allo-HSCT) is another important therapeutic modality for AML patients [5]. However, those with adverse disease risk characteristics and older patients (AML predominantly impacts individuals over the age of 65) are less medically fit for HSCT [6]. Currently, the five-year survival rate for most AML patients remains low [7]. New therapeutic drugs are urgently needed for AML treatment.

AML is characterized by differentiation arrest, as evidenced by a significant increase in the number of immature malignant cells and a decrease in the differentiation of erythrocytes, platelets, and leukocytes. Agents that overcome this block are therapeutically useful in certain AML subtypes, such as acute promyelocytic leukemia (APL) [8,9]. The application of ATRA and arsenic trioxide (ATO) induces the differentiation of abnormal APL cells, resulting in complete remission and overall survival rates of greater than 95% in APL patients [10,11]. However, ATRA is not effective for other myeloid leukemias. There have been extensive efforts to find differentiating drugs or to promote ATRA sensitivity to treat non-APL AML [12].

AML is highly heterogeneous, and many genetic mutations can lead to AML, making it difficult to develop drugs to treat this disease. However, it has been found that HOX genes are commonly upregulated in AML and are directly associated with a poorer prognosis [13]. The HOX family is an evolutionarily highly conserved set of genes encoding DNA-binding transcription factors that were first identified as key regulators of the positional identity along the anterior posterior body axis of animal embryos [14,15,16]. Among all the HOX genes, *HOXA9* is overexpressed in about 70% of AML cases [17]. The overexpression of *HOXA9* in the blood system leads to impaired blood cell differentiation, and transgenic mice overexpressing *Hoxa9* develop myeloid diseases [18]. Using a cellular model of *hoxa9*-enforced myeloid differentiation arrest, studies have identified that the dihydroorotate dehydrogenase (DHODH) inhibitor induces the differentiation of diverse AML subtypes [19]. Several DHODH inhibitors have entered clinical trials for treating AML, supporting the feasibility of using a *Hoxa9* overexpression model to identify pro-differentiation targets for AML treatment [20].

Recently, zebrafish have become a powerful model for studying the pathogenesis and therapeutics of hematological diseases. They have unique advantages, such as a small size, rapid development, and ease of handling, making them suitable for genetic and chemical screening [21,22,23]. There are two distinct waves in zebrafish hematopoiesis, similar to those in higher vertebrate organisms. Primitive hematopoiesis produces myeloid cells and erythrocytes, while definitive hematopoiesis generates hematopoietic stem progenitor cells (HSPCs) [24]. These features have allowed studies on zebrafish blood development to be applied in mammalian systems. Additionally, fish and mammals share a number of genes, signaling pathways, and transcription factors that influence blood cell development and differentiation [25]. For example, *runx1* marks HSPCs in both mice and fish. In differentiated populations, *gata1* regulates the erythroid lineage, while *pu.1* and *c/ebpα* regulate the myeloid lineage, and *ikzf1* labels the lymphoid population [26]. In addition, the zebrafish is a suitable model for large-scale drug screening [27,28,29,30]. As a whole organism, zebrafish screening provides a system to simultaneously address drug toxicity and in vivo drug effects, is a good complement to cell-based screening, and has the potential to discover novel lead compounds.

In this study, we developed a transgenic zebrafish line that overexpresses *hoxa9* in hematopoietic cells under the control of the draculin (*drl*) regulatory element [31]. The transgene Tg(*drl:hoxa9*) also exhibits a strong arrest of myeloid differentiation. Using this line, we screened the Cayman Epigenetic and Prestwick Libraries and found four compounds that were effective in restoring myeloid terminal differentiation in Tg(*drl:hoxa9*), namely ethacrynic acid, khellin, oxcarbazepine, and alendronate. We also examined the effects of these compounds on AML cell differentiation and compared the functions and interactions of ethacrynic acid and ATRA in AML differentiation. Mechanistically, we found that ethacrynic acid and ATRA induced myeloid differentiation by upregulating the IL-17/MAPK signaling pathways. Together, by using Tg(*drl:hoxa9*), our study identified four differentiation-inducing compounds, which may offer novel insights into differentiation therapy for AML.

## 2. Results

### 2.1. Myeloid Differentiation Is Arrested in Tg(drl:hoxa9)

**Figure 1:**
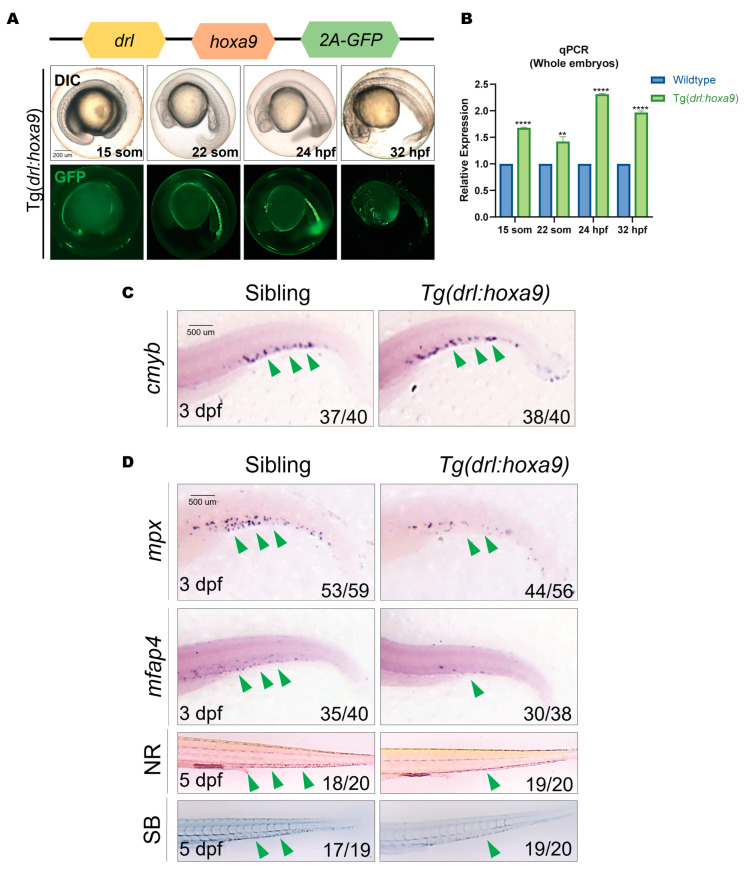
Myeloid development is arrested in Tg(*drl:hoxa9*) zebrafish embryos. (**A**) Schematic representation of the *drl:hoxa9-2A-GFP* reporter gene and the DIC and fluorescent images of Tg(*drl:hoxa9*) embryos. GFP is expressed in the lateral plate mesoderm and derived hematopoietic tissues at different stages. (**B**) RT-qPCR analysis of *hoxa9* expression in Tg(*drl:hoxa9*) embryos compared to sibling embryos. Results are represented as mean ± SEM, *t*-test; ** *p* < 0.01, **** *p*< 0.0001. (**C**,**D**) WISH of *cmyb* (myeloid progenitor cells), *mpx* (granulocytes), and *mfap4* (macrophages) in Tg(*drl:hoxa9*) and sibling embryos at 3 dpf. Neutral red (NR) and Sudan black (SB) staining of macrophages and neutrophils in embryos at 5 dpf. A green arrow indicates the staining signal. *drl*, draculin; WISH, whole mount in situ hybridization; hpf, hours post-fertilization; dpf, days post-fertilization.

We previously generated Tg(*drl:hoxa9-myc*) which overexpresses *hoxa9* in hematopoietic cells under the control of the *drl* element, and this transgene inhibited myeloid differentiation [32]. To obtain an observable transgenic line and select embryos uniformly expressing *hoxa9* to facilitate screening, we generated transgenic lines overexpressing GFP-fused *hoxa9* in hematopoiesis cells using the plasmid *drl-hoxa9-2A-GFP*. In the stable transgenes Tg(*drl:hoxa9*), strong GFP fluorescence was observed in the lateral plate mesoderm and derived tissues during larval development (Figure 1A). We used quantitative reverse transcription PCR (RT-qPCR) to compare the expression levels of *hoxa9* at different development stages in wild-type (WT) and transgenic embryos. The results showed that *hoxa9* was abundantly expressed in Tg embryos (Figure 1B).

We then examined myeloid development by whole mount in situ hybridization (WISH) and histochemical staining. The results showed that myeloid progenitor cells (*cmyb*) did not change in Tg(*drl:hoxa9*) embryos, but myeloid terminally differentiated mature cells, including granulocytes (*mpx* and Sudan black staining) and macrophages (*mfap4* and neutral red staining), were significantly reduced at 3 dpf and 5 dpf (Figure 1C,D), respectively, which was consistent with the results of Tg(*drl:hoxa9-myc*) [32]. Furthermore, we examined the development of erythrocytes at 5 dpf by benzidine staining (Figure S1) and found that erythroid differentiation was not significantly affected in the Tg. These data suggest that this Tg(*drl:hoxa9*) significantly inhibits myeloid immune cell differentiation.

### 2.2. Chemical Screening Identified Chemicals Restoring Myeloid Differentiation in Tg(drl:hoxa9)

**Figure 2:**
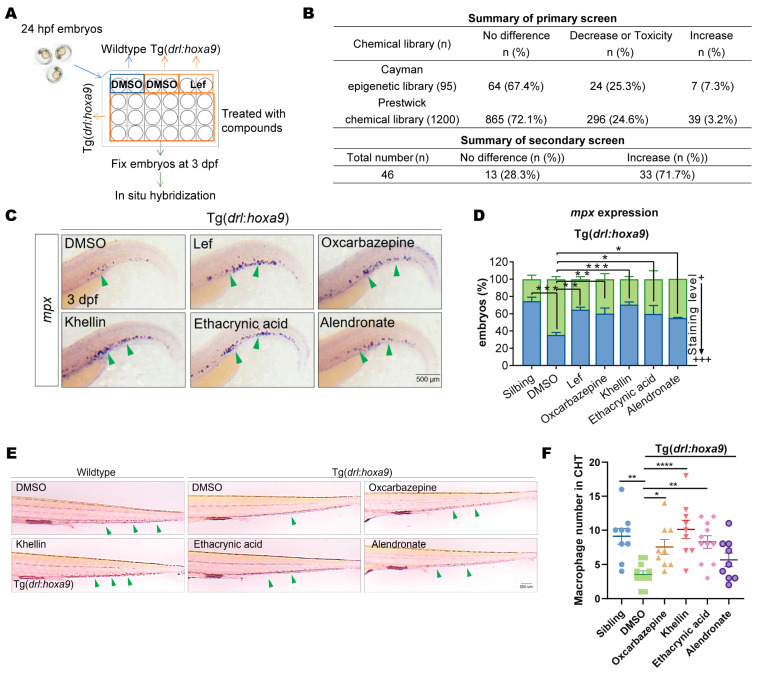
Chemical screening using Tg(*drl:hoxa9*) identified compounds that restored myeloid differentiation. (**A**) A schematic representation of the chemical screening process. The effects of compounds on myeloid differentiation were examined by WISH of *mpx*, and Lef was used as a positive control. (**B**) Summary of primary and secondary screenings. (**C**) Tg(*drl:hoxa9*) embryos were treated with DMSO; Lef; or oxcarbazepine, khellin, ethacrynic acid, and alendronate and then assayed by WISH of *mpx* at 3 dpf. A green arrow indicates the staining signal. (**D**) Quantification of WISH results in (**C**). (**E**) Sibling and Tg(*drl:hoxa9*) embryos were treated with DMSO, Lef, oxcarbazepine, khellin, ethacrynic acid, and alendronate and stained for macrophages with NR at 5 dpf. (**F**) Quantification of results in (**E**). Results in (**D**,**F**) are represented as mean ± SEM, *t*-test; * *p* < 0.05, ** *p* < 0.01, *** *p* < 0.001, **** *p*< 0.0001. NR, neutral red; Lef, leflunomide.

Next, we used Tg(*drl:hoxa9*) embryos to screen for compounds that overcame myeloid differentiation arrest. For this, we first examined the effects of the DHOH inhibitor leflunomide on the Tg. Leflunomide restored myeloid differentiation well in Tg(*drl:hoxa9*), demonstrating the consistency of this Tg with mammalian models for studying myeloid differentiation drugs. We then screened the Prestwick Library (n = 1200, a unique collection of small molecules, 98% of which are FDA- and EMA-approved drugs) and the Cayman Epigenetics Library (n = 95). We treated Tg(*drl:hoxa9*) embryos with compounds at 1 dpf and analyzed the differentiation of granulocytes in the embryos using WISH at 3 dpf (Figure 2A).

Of the 1295 chemicals tested, 72.7% did not alter myeloid differentiation. Meanwhile, 24.7% of the compounds further decreased *mpx* staining in Tg embryos, and many of these compounds affected embryonic development, thereby interfering with granulocyte formation. We found that 2.5% of the compounds (n = 33) increased granulocyte differentiation in Tg(*drl:hoxa9*) (Figure 2B). We further verified the effects of the 33 compounds on myeloid differentiation in a secondary screening and found that oxcarbazepine, khellin, ethacrynic acid, and alendronate significantly restored *mpx* expression in Tg(*drl:hoxa9*), with similar effects as the positive control drug, leflunomide (Figure 2C,D). Moreover, the four drugs recovered macrophage differentiation in Tg(*drl:hoxa9*) embryos (Figure 2E,F). Thus, all four drugs restored the terminal differentiation of myeloid cells in Tg, supporting their ability to induce the differentiation of blocked myeloid cells.

### 2.3. Four Compounds Induce Myeloid Differentiation of AML Cells

**Figure 3:**
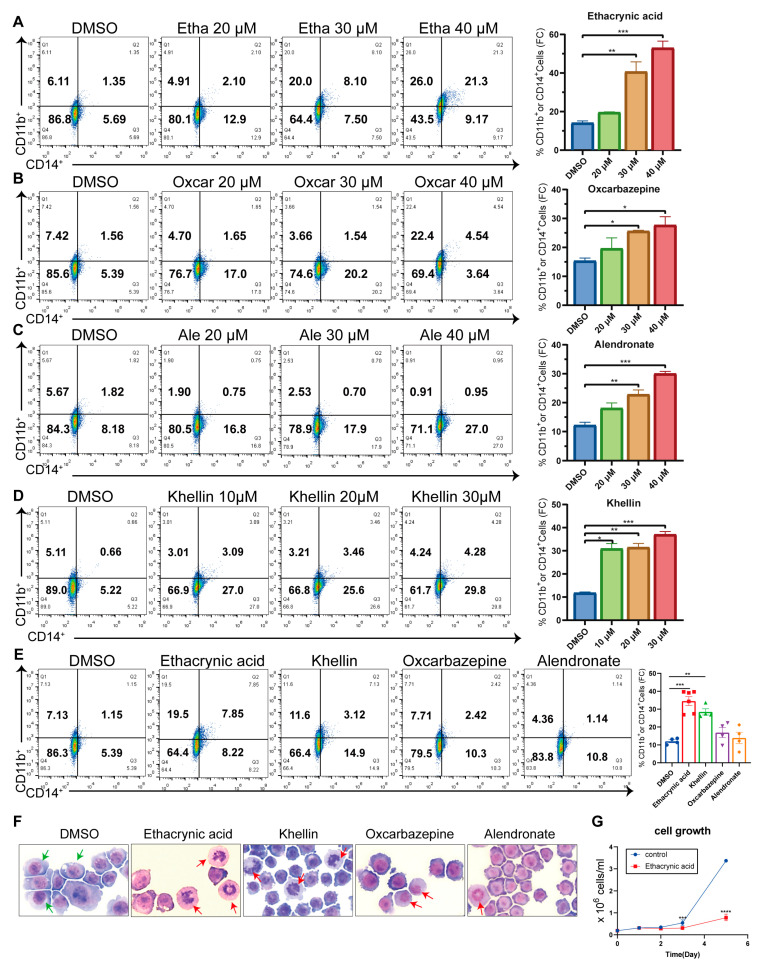
The four identified compounds induce myeloid differentiation of U937 cells. (**A**–**D**) Flow cytometry analysis of CD14 and CD11b expression in U937 cells treated with different doses of ethacrynic acid, oxcarbazepine, alendronate, and khellin. (**E**) Flow cytometry analysis of CD14 and CD11b expression in U937 cells treated with 30 μM ethacrynic acid, oxcarbazepine, alendronate, and khellin for 3 days. (**F**) Representative images of May-Grunwald-Giemsa staining at 200× magnification of U937 cells treated with 30 μM chemicals for 3 days. Green arrows indicate undifferentiated cells and red arrows indicate mature cells. (**G**) The proliferation curve of U937 cells treated with ethacrynic acid (30 μM). Results are represented as mean ± SEM, n = 3, *t*-test; * *p* < 0.05, ** *p* < 0.01, *** *p* < 0.001, **** *p*< 0.0001.

Next, we studied the effects of the four compounds on myeloid differentiation in leukemia cells. For this, we treated U937 cells and HL-60 cells with different doses of drugs and examined differentiation via the expression levels of the surface markers CD11b and CD14 (markers of granulocytes and monocytes, respectively). We found that the four drugs dose-dependently increased the percentage of CD11b- or CD14-positive cells in U937 cells after 72 h of treatment (Figure 3A–D), supporting the ability of all four drugs to induce acute myeloid differentiation. These drugs also induced CD11b or CD14 expression in HL-60 cells, but at lower levels (Figure S2). Therefore, our subsequent studies were mainly performed on U937 cells. In U937 cells, ethacrynic acid and khellin induced differentiation from lower concentrations (20 μM and 10 μM, respectively), and oxcarbazepine and alendronate induced significant differentiation from a higher concentration (30 μM). At the same concentration (30 μM), after 72 h of chemical exposure, CD11b- or CD14-positive cells (as a percentage of the total number of U937 cells) increased from 13.6 ± 3.77 (DMSO) to 39.6 ± 1.24 (ethacrynic acid, *p* < 0.001), 33.5 ± 4.09 (khellin, *p* < 0.01), 20.4 ± 6.91 (oxcarbazepine), and 16.3 ± 3.64 (alendronate) (Figure 3E). To further verify the cellular differentiation, we examined the morphological changes in cells by May-Grunwald-Giemsa staining. As shown in Figure 3F, at a concentration of 30 μM, all four drugs induced chromatin condensation and increased the nucleocytoplasmic ratio in U937 cells, supporting the differentiation of these cells. Among the four drugs, ethacrynic acid and khellin demonstrated better differentiation effects.

In addition, we noticed that the four compounds exhibited different effects on CD11b and CD14 expression (Figure S3). Ethacrynic acid and oxcarbazepine increased the expression levels of CD11b and CD14 (Figure 3A,B and Figure S3). Alendronate induced CD11b expression at a low concentration and induced CD14 expression at high concentrations (Figure 3C and Figure S3). Khellin preferentially induced the expression of CD14 (Figure 3D and Figure S3). Thus, the four compounds had different effects on the differentiation of specific myeloid lineages. We also examined the proliferation of AML cells after ethacrynic acid treatment and found that ethacrynic acid not only induced cell differentiation but also significantly inhibited AML cell proliferation starting from 72 h after treatment (Figure 3G and Figure S3). Thus, the above results suggest that the four chemicals, specifically ethacrynic acid and khellin, induce myeloid differentiation in AML cells.

### 2.4. Ethacrynic Acid Activates IL-17 and MAPK Signaling Pathways during Induction of AML Differentiation

**Figure 4:**
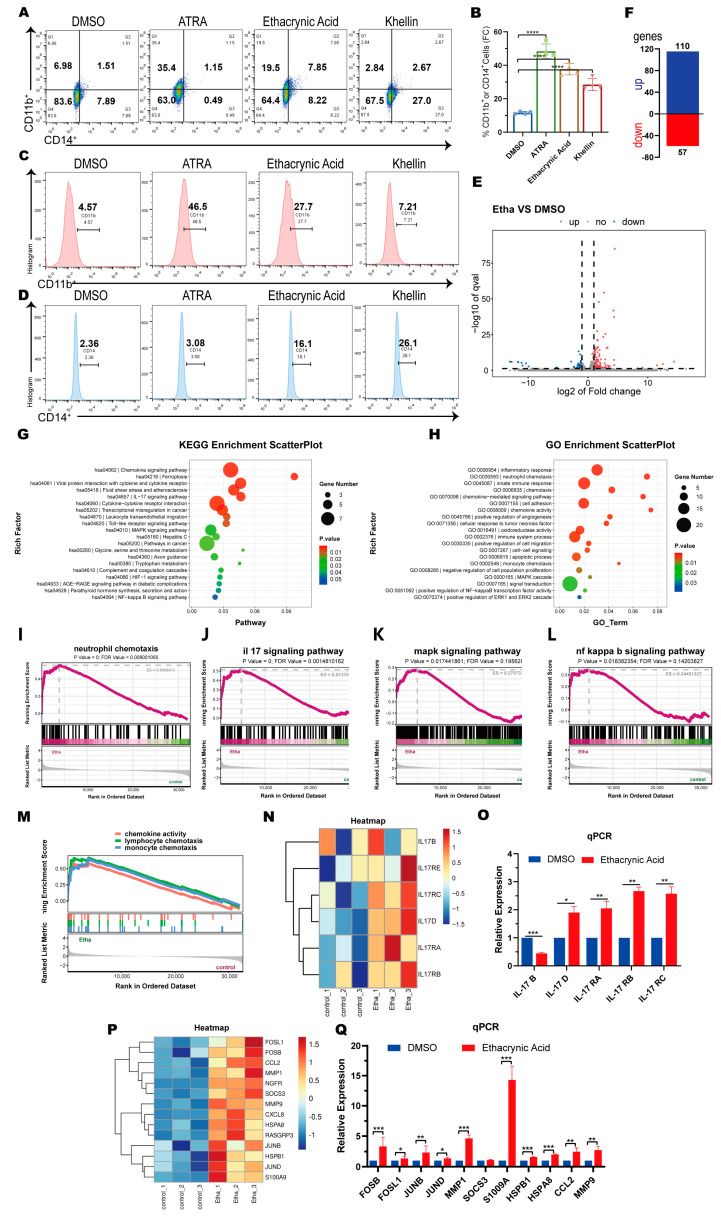
Ethacrynic acid activates the IL-17/MAPK pathways during the induction of AML cell differentiation. (**A**) Flow cytometry analysis of CD14 and CD11b expression of U937 cells treated with ATRA (0.2 μM), ethacrynic acid, and khellin (30 μM) for 3 days. (**B**) Quantitative analysis of results in (A). (**C**,**D**) Flow cytometry analysis of CD1b or CD14 expression of U937 cells treated with ATRA (0.2 μM), ethacrynic acid, and khellin (30 μM) for 3 days. (**E**) Volcano plot of the differential gene expression between DMSO- and ethacrynic-acid-treated groups from three biological replicates. (**F**) Numbers of up- and downregulated genes in the ethacrynic acid group relative to the DMSO group (greater than twofold change, adj. *p* ≤ 0.05). (**G**) GO enrichment analysis of differentially expressed genes. (**H**) KEGG enrichment analysis of differentially expressed genes. (**I**–**M**) GSEA of the expression profile of U937 cells treated with DMSO and ethacrynic acid using various signaling signatures. (**N**) Heatmap of DEGs involved in the IL-17 signaling pathway between the DMSO and ethacrynic acid groups. (**O**) RT-qPCR analysis showing mRNA expression of IL-17 B and D and IL-17 RA, RB, and RC in the ethacrynic acid group compared to the DMSO group. (**P**) Heatmap of DEGs involved in the IL-17/MAPK signaling pathways between the DMSO and ethacrynic acid groups. (**Q**) RT-qPCR analysis showing mRNA expression of FOSB, FOLS1, JUNB, JUND, MMP1, S1009A, HSPB1, HSPA8, CCL2, and MMP9 in the ethacrynic acid group compared to the DMSO group. Results in (**B**,**O**,**Q**) are represented as mean ± SEM, n = 3, *t*-test; * *p* < 0.05, ** *p* < 0.01, *** *p* < 0.001, **** *p*< 0.0001. DEG, differential gene expression.

To further characterize the differentiation abilities of the identified compounds, we compared the effects of ethacrynic acid and khellin, the two drugs with better differentiation capabilities, with ATRA in U937 cells. The FACS analysis showed that ATRA, ethacrynic acid, and khellin all induced robust cell differentiation, and the differentiation percentages of CD11b or CD14 were 46.6% ± 2.2, 37.7% ± 2.3, and 32.5% ± 3.2, respectively (Figure 4A,B). Interestingly, we found that ATRA preferentially induced CD11b expression, and khellin induced CD14 expression, but ethacrynic acid increased both CD11b and CD14 expression and increased CD11b expression more (Figure 4C,D). Thus, the effects of ethacrynic acid on myeloid differentiation may be closer to those of ATRA.

To further investigate the effects of ethacrynic acid on myeloid differentiation, we performed an RNA sequencing analysis for transcriptomic changes in AML cells after ethacrynic acid treatment. We found that 110 genes were upregulated and 57 genes were downregulated in ethacrynic-acid-treated cells (FDR < 0.05 and greater than twofold change, Figure 4E,F). The GO enrichment and KEGG analyses showed that signaling pathways such as inflammatory response, neutrophil chemokine, immune response, chemokine-mediated signaling pathway were enriched in the ethacrynic acid treatment group (Figure 4G,H). In line with this finding, the gene set enrichment analysis (GSEA) revealed positive enrichment in the ethacrynic acid group for the gene signatures with neutrophil chemokine (Figure 4I), NF-κB signaling pathway (Figure 4L), and chemokine activity (Figure 4M). These results together support increased myeloid differentiation and inflammatory responses after ethacrynic acid treatment. More importantly, the results revealed that the IL-17 signaling pathway and MAPK signaling pathway were among the most enriched pathways in the ethacrynic acid group (Figure 4J,K). The differential gene expression (DEG) analysis showed that most of the receptor genes in IL-17 signaling (IL-17 RA, RB, RC, and RD) were upregulated (Figure 4N). MAPK is an important downstream pathway of IL-17. Ten genes in the IL-17/MAPK pathways (FOLS1, FOSB, CCL2, MMP1, NGFR, SOCS3, MMP9, CXCL8, HSPA8, RASGRP3, JUNB, HSPB1, JUND, and S1009A) were also strongly increased (Figure 4P). We verified the expression changes of these genes by RT-qPCR. The results showed that ethacrynic acid significantly increased the expression of IL-17 D, IL-17 RA, RB, RC, FOSB, FOLS1, JUNB, JUND, MMP1, S1009A, HSPB1, HSPA8, CCL2, and MMP9 in U937 cells (Figure 4O,Q). Thus, ethacrynic acid induces myeloid differentiation, closely to ATRA, and activates the IL-17/MAPK signaling pathways during differentiation induction.

### 2.5. Ethacrynic Acid Augments ATRA-Induced Differentiation

**Figure 5:**
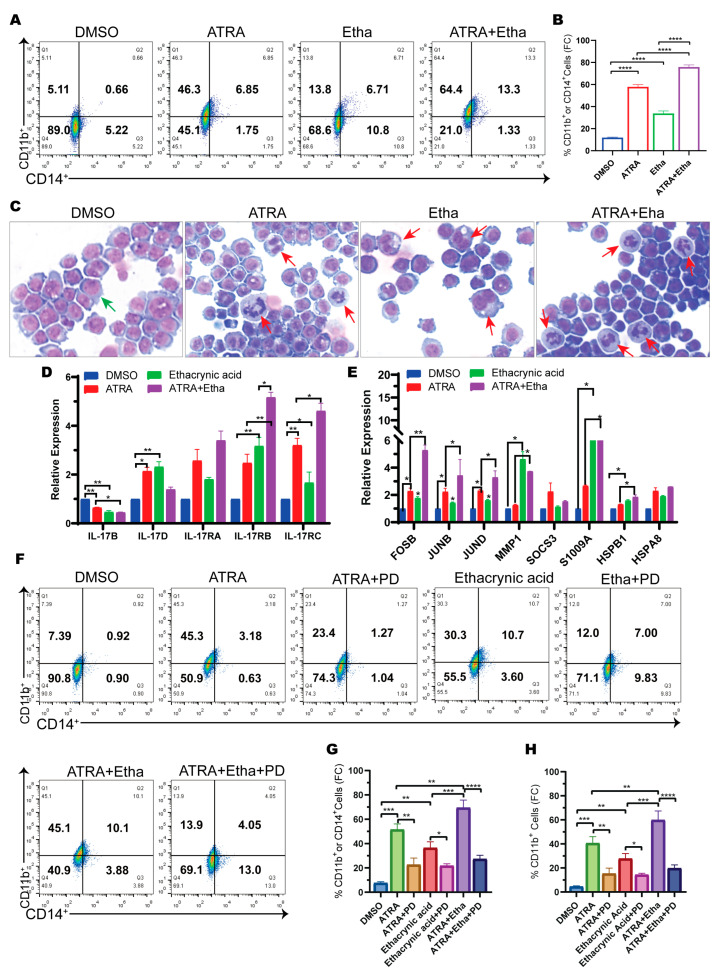
Ethacrynic acid augments ATRA-induced AML differentiation through co-activation of the IL-17/MAPK pathways. (**A**) Flow cytometry analysis of CD14 and CD11b expression of U937 cells treated with ATRA (0.2 μM), ethacrynic acid (30 μM), and an ATRA and ethacrynic acid combination for 3 days. (**B**) Quantitative analysis of results in (**A**). (**C**) Representative images of May-Grunwald-Giemsa staining at 200× magnification of U937 cells treated with ATRA (0.2 μM), ethacrynic acid (30 μM), and an ATRA and ethacrynic acid combination for 3 days. Green arrows indicate undifferentiated cells and red arrows indicate mature cells. (**D**) RT-qPCR analysis of IL-17 (**B**,**D**), RA, RB, and RC in the ATRA, ethacrynic acid, and ATRA plus ethacrynic acid groups compared to the DMSO group. (**E**) RT-qPCR analysis of genes involved in IL-17/MAPK pathways in the ATRA, ethacrynic acid, and ATRA plus ethacrynic acid groups compared to the DMSO group. (**F**) Flow cytometry analysis of CD14 and CD11b expression of U937 cells treated with ATRA (0.2 μM), ethacrynic acid (30 μM), and an ATRA and ethacrynic acid combination with and without PD98059 (20 μM) for 3 days. (**G**) Quantification of results in (**F**). (**H**) Quantification of CD11b-positive cells in each group as in (**F**). Results in (**B**,**D**,**E**,**G**,**H**) are represented as mean ± SEM, n = 3, *t*-test; * *p* < 0.05, ** *p* < 0.01, *** *p* < 0.001, **** *p*< 0.0001.

Previous studies have shown that MAPK signaling is activated during ATRA-induced differentiation [33]. Thus, ethacrynic acid may interact with ATRA to enhance ATRA-induced differentiation and converge on this pathway. To test this idea, U937 cells were treated with the two compounds alone or in combination. The results showed that the percentage of CD11b- or CD14-positive cells increased from 54.9% to 79% after ATRA plus ethacrynic acid treatment compared with ATRA treatment alone (Figure 5A,B). We also analyzed the cell morphological changes after ATRA and ethacrynic acid treatment by May-Grunwald-Giemsa staining. ATRA plus ethacrynic acid co-treatment showed the highest percentage of mature cells (Figure 5C). Thus, the results support that ethacrynic acid promotes ATRA-induced AML differentiation.

We then analyzed the expression changes of genes in the IL-17 and MAPK signaling pathways induced by chemical treatment. The results showed that the expression of IL-17 receptors (IL-17 RA and IL-17 RC) was robustly increased after ATRA treatment (Figure 5D), and that ten genes in the IL-17/MAPK pathways were also significantly upregulated by ATRA. Additionally, the expression of all these genes was further increased after co-treatment (Figure 5E). Thus, both ATRA and ethacrynic acid activated the IL-17/MAPK pathways, and combined treatment further enhanced the activation of this pathway during differentiation (Figure 5F).

To investigate whether IL-17/MAPK pathway activation is required for ATRA- and ethacrynic-acid-mediated differentiation, we used the MAPK inhibitor PD98059 together with ATRA and ethacrynic acid. The results showed that the percentage of CD11b- or CD14-positive cells significantly decreased with the addition of PD98059 to ATRA or ethacrynic acid treatment alone or in combination (Figure 5G). The expression of CD11b or CD14 decreased to the same level in different groups after PD98059 treatment. Additionally, we found that PD98059 predominantly decreased the percentage of CD11b-positive cells (Figure 5H and Figure S4). These results support that MAPK signaling is indeed involved in ATRA- and ethacrynic-acid-induced differentiation, especially in the differentiation of CD11b-positive cells. These data together suggest that ethacrynic acid enhances ATRA-induced myeloid differentiation by co-activating the IL-17/MAPK signaling pathways.

### 2.6. Ethacrynic Acid Is Less Disruptive to Normal Hematopoiesis Than ATRA

**Figure 6:**
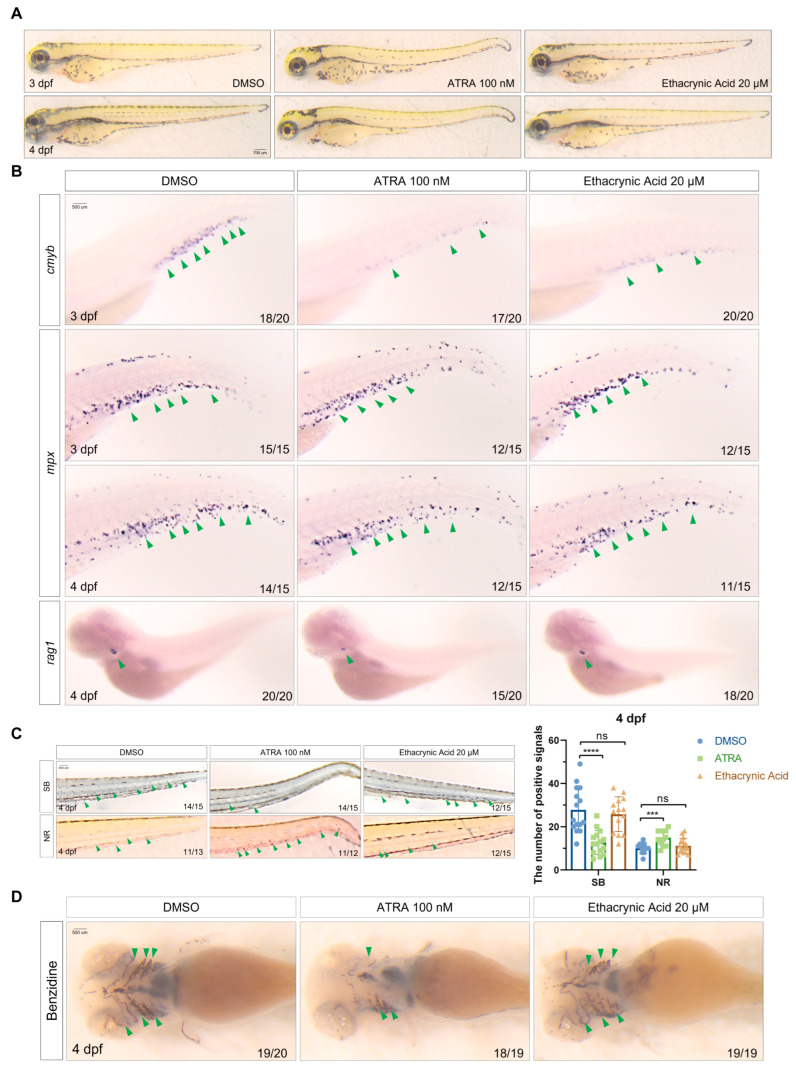
Ethacrynic acid causes less damage to normal hematopoiesis than ATRA. (**A**) DIC images of zebrafish WT embryos at 3 and 4 dpf after treatment with ATRA (0.1 μM) and ethacrynic acid (20 μM) at 24 hpf. (**B**) WISH of *cmyb*, *mpx*, and *rag1* in WT embryos at 3 and 4 dpf after treatment with ATRA and ethacrynic acid at 24 hpf. A green arrow indicates the staining signal. (**C**) SB and NR staining of WT embryos at 4 dpf after chemical treatment. Results are represented as mean ± SEM, n = 3, *t*-test; *** *p* < 0.001, **** *p* < 0.0001; ns, not significant. (**D**) Benzidine staining of WT embryos at 4 dpf after chemical treatment. SB, Sudan black; NR, neutral red.

Although ATRA is effective in treating APL, it often conveys significant toxicities such as severe myelosuppression [34]. We next studied the effects of ethacrynic acid on embryonic development and normal hematopoiesis and compared it to ATRA. For this, we treated wild-type (WT) zebrafish embryos with the two chemicals. In Tg(*drl:hoxa9*) embryos, ethacrynic acid showed the best rescue effect at 20 μM, while ATRA had the best recovery effect at 100 nM (Figure S5). Therefore, we compared the effects of the two drugs at these two concentrations. We found that ATRA is much more toxic than ethacrynic acid and readily causes tail bending and cardiac edema in embryos (Figure 6A). ATRA and ethacrynic acid both inhibited the formation of myeloid progenitor cells (*cmyb*), but the inhibition of ATRA was stronger. ATRA and ethacrynic acid did not significantly affect the development of *mpx*^+^ granulocytes (Figure 6B), but ATRA reduced the Sudan-black-stained neutrophils and increased neutral-red-stained macrophages (Figure 6C). ATRA also significantly inhibited the development of lymphocytes (*rag1*). In contrast, ethacrynic acid did not cause significant changes in these lineages. In addition, ATRA also strongly inhibited erythrocyte development (benzidine staining), whereas ethacrynic acid did not (Figure 6D). The above results suggest that ethacrynic acid is less toxic than ATRA and interferes less with the normal hematopoietic processes in vivo.

## 3. Discussion

AML remains a disease with significant unmet need. Differentiation therapy is emerging as an important treatment modality for AML, but the collection of differentiation-inducing agents is still very limited [35]. Using Tg(*drl:hoxa9*) transgenic zebrafish, we performed a chemical screening and identified oxcarbazepine, khellin, ethacrynic acid, and alendronate as drugs that can alleviate myeloid differentiation arrest in Tg(*drl:hoxa9*). These compounds also induced the differentiation of AML cells. Furthermore, we found that ethacrynic acid enhanced ATRA-induced differentiation by co-activating the IL-17/MAPK signaling pathways. Our study may provide novel insights into differentiation therapy in AML.

Tg(*drl:hoxa9*) transgenic zebrafish exhibit a strong differentiation block of myeloid cells and are a suitable model for screening pro-differentiation compounds. We screened the Prestwick Library’s collection of small molecules, 98% of which are FDA- and EMA-approved drugs, for such compounds. Drug repurposing has recently received immense attention as the process is generally less risky and more cost-effective and can be undertaken in less time [36]. From 1200 drugs, we identified four drugs that induce the differentiation of myeloid cells. All four drugs are in clinical use but have not yet been used to treat AML. We found that the four drugs restored myeloid differentiation in Tg(*drl:hoxa9*), but they showed different effects in promoting the maturation of specific lineages in AML cells. ATRA induced CD11b expression in U937 and HL-60 cells, consistent with previous studies [37]. Ethacrynic acid increased the expression of CD11b and CD14, with a higher increase in that of CD11b, and the ATRA–ethacrynic acid combination further increased CD11b expression and the dual expression of CD11b and CD14. Khellin and alendronate preferentially induced CD14. Oxcarbazepine, on the other hand, induced CD11b at lower concentrations and CD14 at higher concentrations. The induction of CD11b is associated with granulocytic differentiation [38], whereas CD14 induction and the simultaneous induction of CD14 and CD11b are associated with monocytic differentiation [39,40]. The lineage-specific differentiation effects of drugs are largely determined by two factors: the lineage potential of AML cells and the signaling pathways induced by the individual drug. The specific effects and associated mechanisms of these drugs deserve further detailed study in the future. In addition, AML is a highly heterogenous disease, and the AML-like model used in our study is not representative of the broad human AML family. Future studies also need to examine the effects of these drugs on more AML models. Furthermore, we used relatively high doses (>20 μM) to treat the AML cells, which may result in potential off-target effects. Future studies will need to examine these potential issues as well.

Ethacrynic acid is used in clinical practice to promote the excretion of excess water and salts from the body in urine. It is one of the most used loop diuretics, acting by inhibiting the Na^+^–K^+^–2Cl_2_ symporter in the kidneys [41,42,43]. It has also been reported to act as a glutathione S-transferase P1-1 (GSTP1-1) [44] inhibitor and a WNT inhibitor [45] and exhibit anti-tumor effects in a range of cancer cells. It can directly inhibit cell proliferation or induce apoptosis on tumor cells or enhance the cytotoxicity of anti-tumor agents [46,47,48,49]. We found that ethacrynic acid induces the differentiation and inhibits the proliferation of AML cells. We also found that ethacrynic acid enhances the differentiation effects of ATRA, and a combination of these two drugs has additive effects in inducing AML differentiation. A recent study by Li et al. showed that the combination of ethacrynic acid and ATRA could have profound synergistic differentiation-inducing effects in AML cells [50]. Our studies support the view that the combinatorial use of ATRA and ethacrynic acid may benefit AML therapy. In that study, Li et al. also reported that ethacrynic acid and ATRA induced differentiation by the reactive oxygen species (ROS)-MEK/ERK and ROS-Akt pathways. However, they also showed that NAC, an antioxidant, decreased ROS levels thoroughly in AML cells but only partially inhibited ethacrynic-acid- and ATRA-triggered differentiation. Therefore, besides ROS signaling, other signals may participate in ethacrynic-acid- and ATRA-triggered differentiation. By RNA sequencing, we found that the IL-17 pathway was significantly upregulated after ethacrynic acid treatment. IL-17 is known to stimulate granulopoiesis in mice and humans [51]. It also stimulates myeloid development in zebrafish [52]. IL-17 can activate downstream signaling pathways such as MAPKs to induce gene expression of pro-inflammatory chemokines and cytokines. After ethacrynic acid treatment, the MAPK signaling pathway was significantly increased. MAPK pathways are known to be involved in ATRA-induced differentiation [53,54]. We found that both IL-17 and MAPK signaling was upregulated by ATRA treatment, and their expression was further upregulated by combined ethacrynic acid and ATRA treatment. Furthermore, administration of the MAPK inhibitor nearly completely diminished ethacrynic-acid-induced as well as ATRA and ethacrynic acid co-induced CD11b differentiation. Thus, ethacrynic acid and ATRA induction of granulocyte differentiation is dependent on the IL-17/MAPK pathways as well as ROS pathways. Convergence of these signaling pathways may help to amplify the cooperative induction of AML cells by ATRA and ethacrynic acid. A more detailed analysis of the complexity underlying the cooperation between ATRA and ethacrynic acid during differentiation will contribute to broadening the clinical applications of the drug combination in AML therapy.

Although ATRA is highly effective in inducing cellular differentiation in APL, it also has many side effects, including cardiac toxicity, respiratory distress, and severe myelosuppression [55]. ATRA plays a complex role in normal hematopoiesis. About 14–16% of APL patients after administration of ATRA develop RAS, which is an unpredictable but frequent complication with an associated mortality of about 2% [56]. In addition, ATRA has a short half-life in the body and requires frequent and high-dose administration. These factors all limit the clinical use of ATRA. Ethacrynic acid also has side effects such as ototoxicity and gastrointestinal symptoms. In our studies, we found that ethacrynic acid is less toxic to embryos than ATRA. We also found that ethacrynic acid interferes less with normal hematopoiesis compared to ATRA. Thus, the ethacrynic acid and ATRA combination could probably reduce the side effects and decrease the risk of developing RAS, leading to better prospects in clinical use.

Collectively, using zebrafish Tg(*drl:hoxa9*) embryos to screen the FDA-approved drug library, we uncovered four drugs that induce the terminal differentiation of myeloid cells that are blocked from differentiation. We also revealed that ethacrynic acid enhances ATRA-induced differentiation in AML cells partly through the IL-17/MAPK pathways. Our studies support that the combined use of ethacrynic acid and ATRA may be an effective differentiation therapy with better prospects for AML.

## 4. Materials and Methods

### 4.1. Zebrafish Maintenance and Embryo Handling

The wild-type zebrafish (*Danio rerio*) line was purchased from the China Zebrafish Resource Center (CZRC, Wuhan, China). The adults were raised in a circulating water system under a 14 h/10 h light/dark cycle at 26–28 °C and fed two times per day. During the experimental period, the volume pH ranged from 7.8 to 7.9. The dissolved oxygen ranged from 6.95 to 7.23 mg/L. The water temperature ranged from 26.5 to 28 °C. The embryos were collected and kept at 28.5 °C in egg water or E3 medium (5 mM NaCl, 0.17 mM KCl, 0.33 mM CaCl_2_, and 0.33 mM MgSO_4_) with a density of 100 embryos per 10 cm diameter Petri dish. Embryos were staged by hours post-fertilization (hpf) and days post-fertilization (dpf). The fish were maintained, handled, and bred according to standard protocols (202301238) from the Institutional Animal Care Committee of Shanghai Jiao Tong University.

### 4.2. Generation of hoxa9 Overexpression Lines

To overexpress *hoxa9* in the hematopoietic system, we injected the plasmid (20 ng/μL) driven by the *drl* regulatory element containing the full length of the zebrafish *hoxa9* or *hoxb4* sequence and the 2AGFP sequence and Tol2 mRNA (150 ng/μL) into WT embryos at the one-cell stage. After 24 hpf, injected embryos showing a positive expression of GFP were raised to adults (F0 founder). F0 fish were outcrossed to WT fish, and GFP-positive embryos were raised to adults (F1). F1 fish were outcrossed to WT fish again to obtain the final stable transgenic lines.

### 4.3. Gene Expression Analysis by Real-Time qPCR (RT-qPCR)

Gene expression was evaluated using RT-qPCR. Briefly, the total RNA was extracted from embryos with Trizol reagent (R0016, Beyotime, Shanghai, China). The cDNAs were synthesized from the total RNA using the Hifair^®^ II 1st Strand cDNA Synthesis Super Mix (11123ES60, Yeasen, Shanghai, China). The Hieff^®^ qPCR SYBR Green Master Mix (11203ES08, Yeasen, Shanghai, China) was used for qPCR analysis. Each target gene was calculated using the 2^−ΔΔCt^ method. The primers for different target genes and β-actin (the reference gene) are listed in Supplementary Table S1.

### 4.4. Whole Mount In Situ Hybridization (WISH)

A standard protocol [57] was followed for preparing antisense digoxigenin-UTP labeled RNA probes (*cmyb*, *mpx*, and *mfap4*). Embryos at the desired time point were fixed overnight in 4% paraformaldehyde (PFA) at 4 °C, bleached, and dehydrated in methanol at −20 °C for at least two hours. Further processing of the embryos was conducted according to the previously published protocol [57]. For *cmyb^+^*, *mpx^+^*, and *mfap4^+^* staining levels, we selected the CHT region to estimate the signal areas. The stained embryos were imaged under an SZX16 stereomicroscope or BX53 microscope (Olympus, Tokyo, Japan).

To calculate the WISH staining results, the embryos were divided into two groups according to the staining level: a strong staining group and a weak staining group. For statistical analysis, groups with strong staining were used in the *t*-test.

### 4.5. Neutral Red Staining and Benzidine Staining

Embryos were treated with 0.003% N-phenylthiourea PTU (Sigma-Aldrich, St. Louis, MO, USA) before 24 hpf to prevent pigment formation. Optimal staining of macrophages in live embryos was obtained by incubating embryos in 2.5 μg/mL neutral red/E3 media (A600652, Sangon Biotech, Shanghai, China) at 28.5 °C in the dark for 6 h according to the procedures in previous studies [58]. 

For benzidine staining, embryos were incubated in benzidine staining solution (2 mL 5 mg/mL benzidine stock prepared in methanol, 4.966 mL ddH_2_O, 33.4 µL 3 M NaOAC, and 200 µL 30% H_2_O_2_) for 30 min in the dark. After washing with PBST (PBS containing 1% Tween20), embryos were fixed in 4% PFA overnight and washed. The stained embryos were imaged under an SZX16 stereomicroscope.

### 4.6. Sudan Black Staining

Sudan black staining was performed according to [59] with some modifications. Embryos were fixed in 4% PFA at 4 °C for overnight and washed three times in PBS. Embryos were then stained with Sudan black solution (0.18% stock diluted 1:5 in 70% ethanol and 0.1% phenol) for 30 min to 5 h. A stereomicroscope was used to check if neutrophil granules had taken up the stain. Next, a series of 5 min washes were performed starting with a single wash in 70% ethanol followed by a single wash with a 1:1 ratio of 70% ethanol and PBS, followed by a final wash in PBS. The stained embryos were imaged under the SZX16 stereomicroscope.

### 4.7. Drug Treatment of Embryos

The Cayman Epigenetic Library (Cayman Chemical 11076), and Prestwick Library (Greenpharma, Orléans, France) were used in this screen. Ethacrynic acid (HY-B1640), khellin (HY-B1394), oxcarbazepine (HY-BO114), alendronate (HY-B11101), and leflunomide (HY-B0083) were purchased from MedChemExpress (Monmouth Junction, NJ, USA). All these drugs were dissolved in dimethyl sulfoxide (DMSO) to prepare a stock solution, except for alendronate, which was dissolved in H_2_O. For the treatments, the stock solution was diluted in egg water (E3 medium) until reaching the working concentrations (5–40 µM). In the chemical screening, the compound from the libraries was examined at 33 μM. Leflunomide is a positive drug. We divided Tg(*drl:hoxa9*) embryos into 24-well plates (20 embryos per group per experiment) and treated the embryos at 1 dpf; after 48 h of chemical treatment, we analyzed the differentiation of granulocytes in the embryos at 3 dpf using WISH.

### 4.8. Cell Culture and Chemical Treatment

U937 and HL-60 cells were purchased from the Chinese Academy of Sciences Cell Bank. U937 cells were maintained in RPMI 1640 medium (HyClone, Thermo Fisher Scientific, Waltham, MA, USA) supplemented with 10% fetal calf serum (FBS, Gibco, Grand Island, NY, USA). HL-60 cells were maintained in IMDM medium (HyClone, Thermo Fisher Scientific, Waltham, MA, USA) supplemented with 20% fetal calf serum (FBS, Gibco, NY, USA). All cells were cultured in a 5% CO_2_ humidified atmosphere at 37 °C. The cells were treated with DMSO or the different chemicals at the indicated doses for 3 days and then analyzed for changes in cell differentiation or gene expression.

### 4.9. Cell Differentiation Assays

To evaluate cell differentiation, morphology and CD11b and CD14 expression were detected. The expression of the cell surface differentiation-related antigens CD14 and CD11b was determined by flow cytometry. Fluorochrome-labeled anti-human CD14/FITC and anti-human CD11b/PE antibodies were purchased from BioLegend (San Diego, CA, USA). After the desired treatment, U937 cells were suspended in FACS buffer (1X PBS containing 1% FBS) and stained for 1 h at room temperature in the dark. The data were collected on LSRFortessa (Becton, Dickinson and Company, Franklin Lakes, NJ, USA). Morphology was determined using May-Grunwald-Giemsa staining (Sigma-Aldrich, St. Louis, MO, USA) according to the manufacturer’s protocol. The cells were centrifuged into slides by cytospin (Sigma Biomolecules, St. Louis, MO, USA) and viewed at ×200 magnification.

### 4.10. Cell Proliferation Assay

Cell growth was assessed using a Cell Counting Kit 8 (K1018, APEXBIO, Houston, TX, USA). First, 10 µL CCK-8 solution was added to 96-well plates at the time specified in the manufacturer’s instructions, and then 3 × 10^3^ cells were seeded in each well in advance. Cell proliferation was measured by absorbance at 450 nm after continued incubation for 4 h at 37 °C. All experiments were repeated three times.

### 4.11. Analysis of Genome-Wide Transcription Changes in U937 Cells

To evaluate genome-wide gene expression changes induced by ethacrynic acid, 1.5 × 10^6^ U937 cells were treated for 3 days, and the total RNA was extracted using Trizol, with 500 ng used to construct a sequencing library. Three biological replicates were used for both the ethacrynic acid and DMSO groups. The libraries were quality-checked and quantified by LC-Bio Technology Company (Hangzhou, China). We performed 2 × 150 bp paired-end sequencing (PE150) on an Illumina Novaseq 6000 system (Illumina, San Diego, CA, USA) following the vendor’s recommended protocol. 

### 4.12. RNA-Seq Analysis

Reads obtained from the sequencing machines were further filtered by Cutadapt (version:1.9) to obtain high-quality clean reads. We used the HISAT2 (version:2.0.4) package to map reads to the reference genome of Homo sapiens GRCh38. The mapped reads of each sample were assembled using StringTie (version 1.3.4d) with default parameters. Then, all transcriptomes from all samples were merged to reconstruct a comprehensive transcriptome using gffcompare software (version 0.9.8). After the final transcriptome was generated, StringTie (version 1.3.4d) was used to estimate the expression levels of all transcripts. StringTie and ballgown were used to determine the expression level for mRNAs by calculating the FPKM (FPKM = [total-exon-fragments/mapped-reads (millions) × exon-length (kB)]). Differentially expressed mRNAs were selected with fold change >2 or fold change <0.5 and with a parametric F-test comparing nested linear models (*p*-value < 0.05) by the R package edgeR (https://bioconductor.org/packages/release/bioc/html/edgeR.html (accessed on 18 June 2024)). Differentially expressed genes were then subjected to enrichment analysis of GO functions and KEGG pathways.

### 4.13. Statistical Analysis

For WISH and RT-qPCR analyses, data from all triplicate experiments were reported as mean values ± SEM. Statistical analyses were performed using GraphPad Prism 8.0.2 (GraphPad Software, San Diego, CA, USA) using the *t*-test. The statistical significance is marked as “ns” in the case of no statistical significance, * for *p* < 0.05, ** for *p* < 0.01, *** for *p* < 0.001, and **** for *p* < 0.0001.

## Data Availability

Raw data from the RNA-seq analysis have been deposited in NCBI Gene Expression Omnibus (GEO) under review.

## Supplementary Materials

The following supporting information can be downloaded at: https://www.mdpi.com/article/10.3390/ijms25147798/s1.
