# Supplementary material for: In Vivo Chemical Screening in Zebrafish Embryos Identified FDA-Approved Drugs That Induce Differentiation of Acute Myeloid Leukemia Cells
Source: Int J Mol Sci. 2024 Jul 16;25(14):7798. doi: 10.3390/ijms25147798 (PMC11277044; doi:10.3390/ijms25147798)
Supplement: Supplementary file 1 [file ijms-25-07798-s001.zip › ijms-3032134-supplementary.pdf]

Supplementary Figures

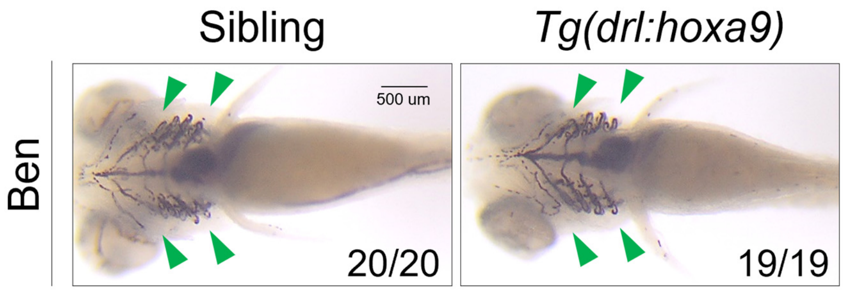

Figure S1. Benzidine staining for hemoglobin in Tg(*drl:hoxa9*) embryos at 5 dpf. A green arrow indicates the staining signal.

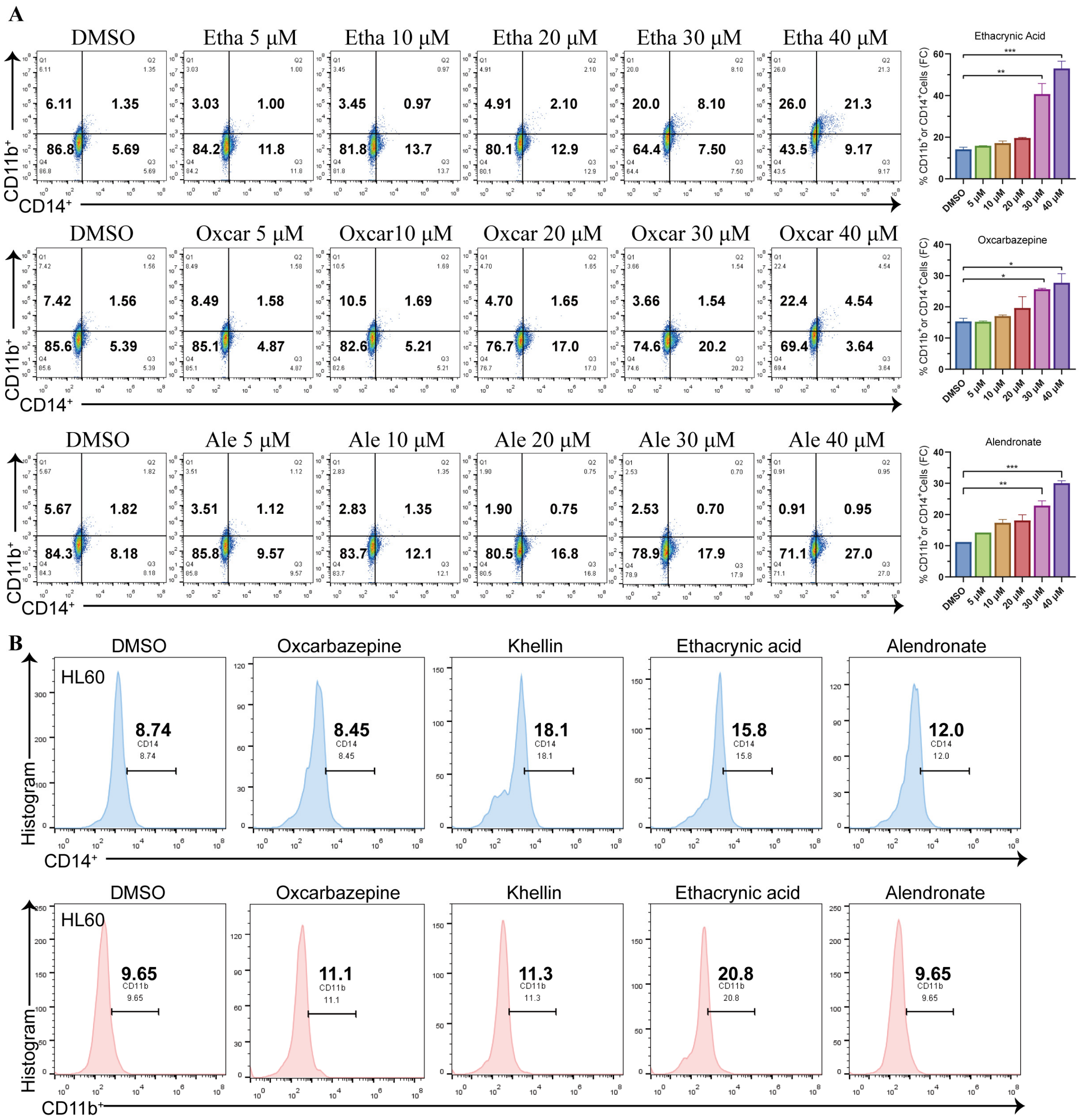

Figure S2. The four identified compounds induced cellular differentiation in U937 and HL60 cells. (A) Flow cytometry analysis of CD14 and CD11b expression of U937 cells treated with different dose of ethacrynic acid, oxcarbazepine and alendronate. T-test, mean ± SEM, n=3, t-test, *P < 0.05, **P < 0.01, ***P < 0.001. (B) Flow cytometry analysis of CD14 and CD11b expression of HL-60 cells treated with ethacrynic acid, khellin, oxcarbazepine and alendronate (30 μM) for 3 days.

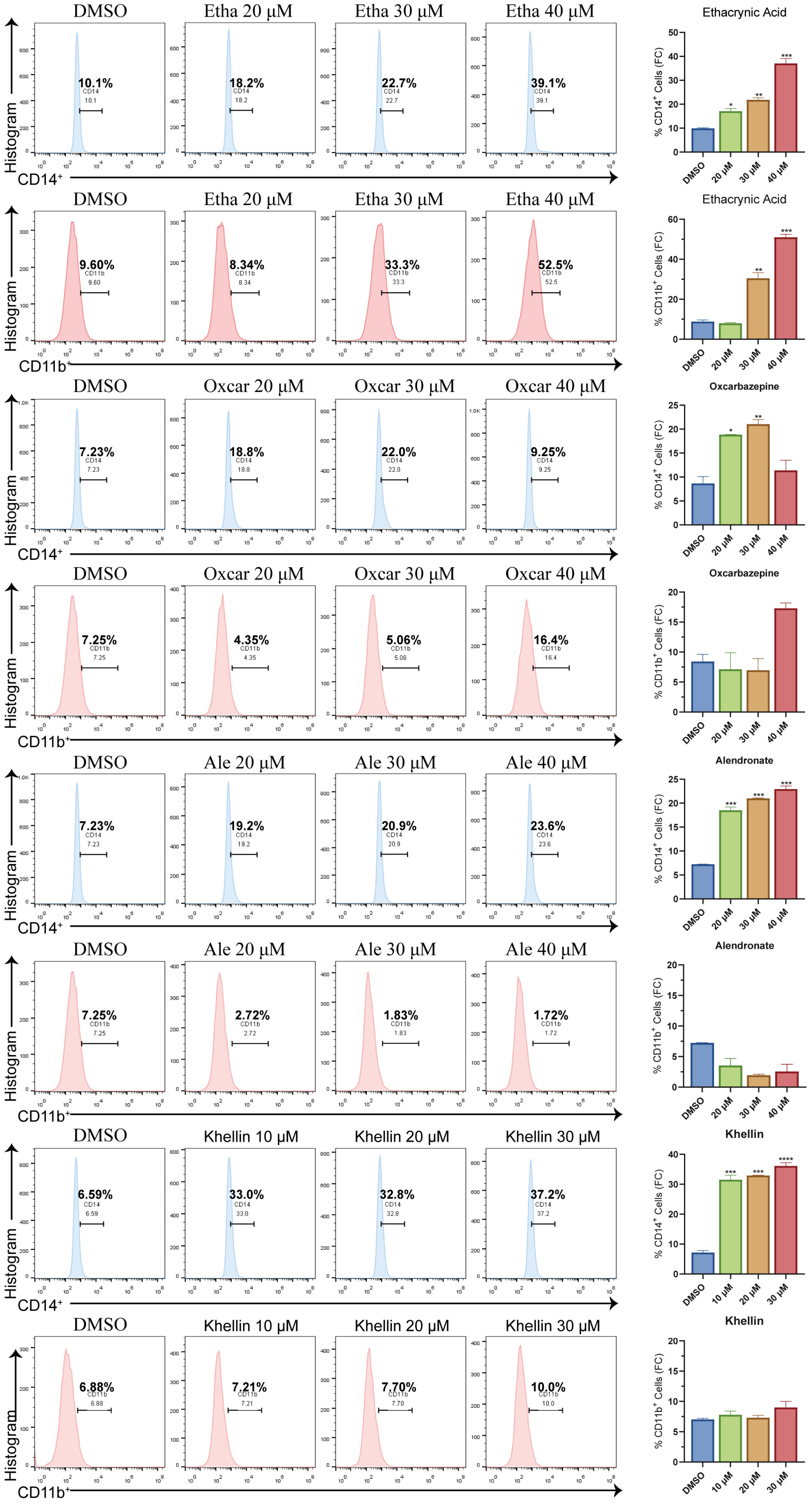
DMSO
Etha 20 µM
Etha 30 µM
Etha 40 µM
10.1%
18.2%
22.7%
39.1%
Histogram
CD14⁺
Ethacrynic Acid
% CD14⁺ Cells (FC)
DMSO
20 µM
30 µM
40 µM
9.60%
8.34%
33.3%
52.5%
CD11b⁺
% CD11b⁺ Cells (FC)
Oxcar 20 µM
Oxcar 30 µM
Oxcar 40 µM
7.23%
18.8%
22.0%
9.25%
Oxcarbazepine
7.25%
4.35%
5.06%
16.4%
Ale 20 µM
Ale 30 µM
Ale 40 µM
7.23%
19.2%
20.9%
23.6%
Alendronate
7.25%
2.72%
1.83%
1.72%
Khellin 10 µM
Khellin 20 µM
Khellin 30 µM
6.59%
33.0%
32.8%
37.2%
Khellin
10 µM
6.88%
7.21%
7.70%
10.0%

Figure S3. Flow cytometry analysis of CD11b or CD14 expression, respectively, of U937 cells treated with ethacrynic acid (30 μM), oxcarbazepine (30 μM), alendronate (30 μM) and khellin (30 μM) for 3 days. T-test, mean ± SEM, n=3, t-test, *P < 0.05, **P < 0.01, ***P < 0.001.

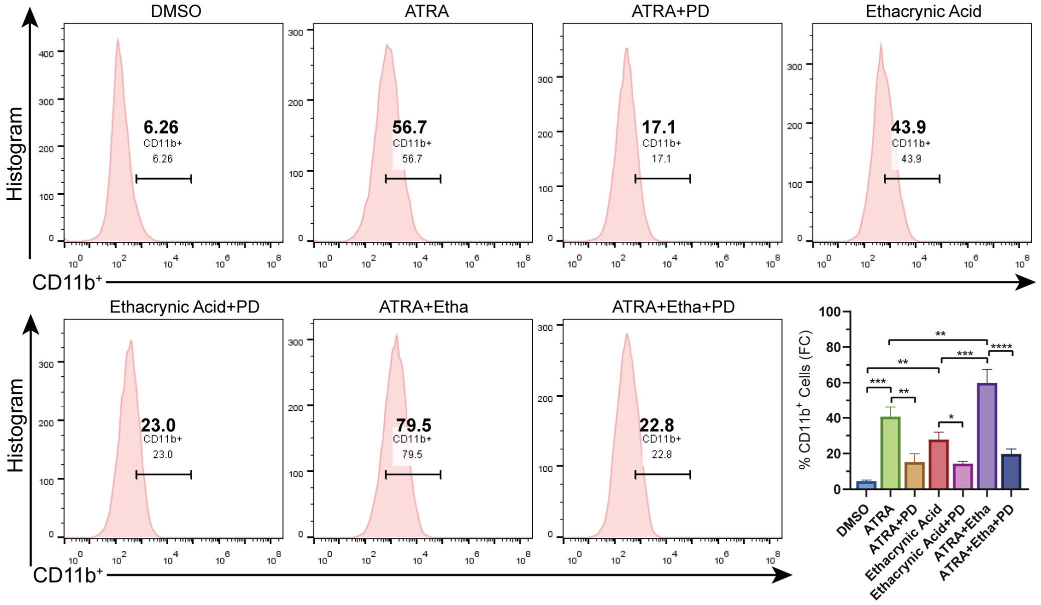

Figure S4. Flow cytometry analysis of CD11b expression of U937 cells treated with ATRA (0.2 μM), ethacrynic acid (30 μM) and the combination of ATRA and ethacrynic acid with and without PD98059 (20 μM) for 3 days. T-test, mean ± SEM, n=3, t-test, *P < 0.05, **P < 0.01, ***P < 0.001, ****p< 0.0001.

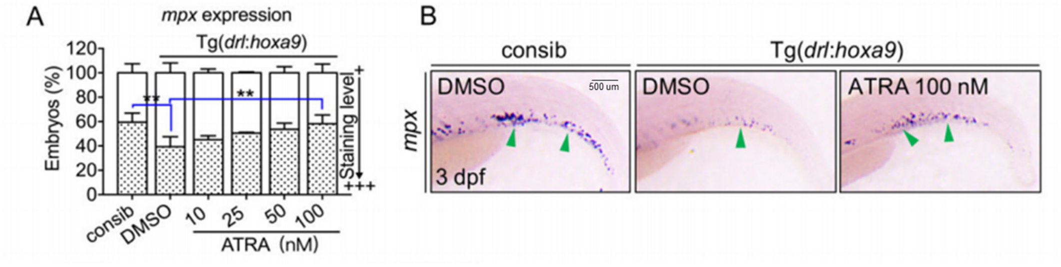

Figure S5. 100 nM of ATRA restores myeloid differentiation in Tg(*drl:hoxa9*) zebrafish embryos. A green arrow indicates the staining signal. T-test, mean ± SEM, n=3, t-test, **P < 0.01.

Table S1. RT-qPCR primers.

Primer	Sequence
zhoxa9-F	TCAGTATGTCAGCCCCATGC
zhoxa9-R	GATTAACCGTCGGTGGCTCA
zhoxb4-F	TCCCAGAGCGACTACCTACC
zhoxb4-R	GACCCTGGCACGATGAGTAG
hIL-17 RA-F	AGACACTCCAGAACCAATTCC
hIL-17 RA-R	TCTTAG AGTTGCTCTCCACCA
hIL-17 RC-F	ACCAGAACCTCTGGCAAGC

hIL-17 RC-R	GAGCTGTTCACCTGAACACA
hIL-17 B-F	GCCCGCATGGAGGAGTATGAG
hIL-17 B-R	TTCACACAGCCCAGACACAGG
hIL-17 RB-F	CAGCGACTGCATCCGACATAAAGG
hIL-17 RB-R	TTCTTGATCCTTTCGTGCCTCCAC
hIL-17 D-F	GCGTGCTCAGTGCCTTCCAC
hIL-17 D-R	ACGTCCTCCTCGCCGAACAG
hFOSB-F	ACCCTCTGCCGAGTCTCAATAT
hFOSB-R	GCCACTGCTGTAGCCACT
hFOSL1-F	CTCCAGGGGTACGTCGAAG
hFOSL1-R	TCAGTTCCTTCCTCCGGTTC
hJUNB-F	CCCTGGACGATCTGCACAAG
hJUNB-R	GAGTAGCTGCTGAGGTTGGTG
hJUND-F	TTTGGAAGAGAGAAGAACAGAG
hJUND-R	CCAAGGATTACAAACAGGAATG
hMMP1-F	TGTGGTGTCTCACAGCTTCC
hMMP1-R	CAACTTGCCTCCCATCATTCT
hSOCS3-F	CAGTACGATGCCCCGCTTTA
hSOCS3-R	TCAGGTGGTACTCCCCCTTC
hS1009A-F	ATCAACACCTTCCACCAATACTCTG
hS1009A-R	AGGTTAGCCTCGCCATCAGC
hHSPB1-F	AGCTGACGGTCAAGACCAAG
hHSPB1-R	TGGGATGGTGATCTCGTTGG
hHSPA8-F	TTGCTGCTCTTGGATGTC
hHSPA8-R	TGTGTCTGCTTGGTAGGA
hCCL2-F	AGAATCACCAGCAGCAAGTGTCC
hCCL2-R	TCCTGAACCCACTTCTGCTTGG
hMMP9-F	TTGACAGCGACAAGAAGTGG
hMMP9-R	GCCATTCACGTCGTCCTTAT
